# LDCT screening results among eligible and ineligible screening candidates in preventive health check-ups population: a real world study in West China

**DOI:** 10.1038/s41598-024-55475-x

**Published:** 2024-02-28

**Authors:** Ting Bao, Bingqing Liu, Ruicen Li, Zhenzhen Li, Guiyi Ji, Youjuan Wang, Hanwei Yang, Weimin Li, Wenxia Huang, Yan Huang, Huairong Tang

**Affiliations:** 1 Health Management Center, General Practice Medical Center, West China Hospital, Sichuan University, Chengdu, 610041 China; 2 Translational Informatics Center, Institutes for Systems Genetics, Frontiers Science Center for Disease-Related Molecular Network, West China Hospital, Sichuan University, Chengdu, 610212 China; 3 West China School of Public Health, Department of Epidemiology and Health Statistics, Sichuan University, Chengdu, 610041 China; 4 Department of Pulmonary and Critical Care Medicine, Sichuan University West China Hospital, Chengdu, 610041 China

**Keywords:** Lung cancer screening, Low-dose spiral CT, Ineligible screening candidates, False-positive biopsy, Outcomes research, Cancer screening

## Abstract

To compare the LDCT screening results between eligible and ineligible screening candidates in preventive health check-ups population. Using a real-world LDCT screening results among people who took yearly health check-up in health management center of West China Hospital between 2006 and 2017. Objects were classified according to the China National Lung Cancer Screening Guideline with Low-dose Computed Tomography (2018 version) eligibility criteria. Descriptive analysis were performed between eligible and ineligible screening candidates. The proportion of ineligible screening candidates was 64.13% (10,259), and among them there were 4005 (39.04%) subjects with positive screenings, 80 cases had a surgical lung biopsy. Pathology results from lung biopsy revealed 154 cancers (true-positive) and 26 benign results (false-positive), the surgical false-positive biopsy rate was 4.17%, and ineligible group (7.69%) was higher than eligible group (2.47%), *P* < 0.05. Further, in ineligible screening candidates, the proportion of current smokers was higher among males compared to females (53.85% vs. 4.88%, *P* < 0.05). Of the 69 lung cancer patients detected in ineligible screening candidates, lung adenocarcinoma accounts for a high proportion of lung cancers both in male (75.00%) and female (85.00%). The proportion of ineligible screening candidates and the surgical false-positive biopsy rate in ineligible candidates were both high in health check-ups population.

## Introduction

The incidence and mortality of lung cancer rank first among cancers in China^1^. According to statistics from the International Organization for Cancer Research, the number of lung cancer cases in China had exceeded 774,000, and the number of lung cancer deaths had exceeded 690,000 in 2018^2^. In comparison to the previous reports in 2015, the incidence and mortality of lung cancer have increased by 5.6% and 13.1% respectively^1,2^. Mortality of lung cancer can be decreased by early screening and early diagnosis effectively. Since 2011, the National Lung Screening Trial (NLST) in the United States confirmed that low-dose computed tomography (LDCT) screening for smokers at high risk of lung cancer can effectively reduce lung cancer mortality^3^, professional organizations have issued guidelines recommending high-risk lung cancer candidates^4–7^. Recently, a growing number of researches have indicated that the risks and benefits of lung cancer screening will need to be weighed if incorporating into real world practices^8,9^. Screening high-risk lung cancer individuals is most effective. However, previous studies have shown that an estimated 40–60% of lung cancer patients do not meet the United States Preventive Task Force (USPTF) criteria derived from the NLST eligibility criteria^10,11^. In the 2019 Behavioral Risk Factor Surveillance System Survey, only 20.9% met all screening eligibility criteria^12^. What is obviously different from the NLST screening technology plan, the definition of high-risk groups for lung cancer in China is more flexible. This includes^13–15^ selecting the starting age based on lung cancer incidence data in each region; smoking ≥ 20 packs per year; other important risk factors in each region can also be used as conditions for screening high-risk groups. This is related to the actual situation of large differences in^16^. Our previous study had found that missed diagnosis rate of lung cancer was high in ineligible candidates for preventive health check-ups according to current guidelines^17^. The conclusion still remains controversial, it still warrants further analysis. More and more health check-up institutions have listed LDCT as a routine screening item, which has resulted in most of the subjects who do not meet the LDCT screening criteria. In the real world, whether the application of LDCT screening in non-high-risk populations has more advantages or disadvantages is still unknown. Also, the characteristics of those individuals who were screened despite not meeting eligibility criteria were not known. Consequently, to evaluate the difference of LDCT screening results between eligible screening candidates and ineligible screening candidates in preventive health check-ups population is necessary.

## Results

### Baseline characteristics

A total of 15,996 people participated in the LDCT baseline screening, with an average age of 50.3 ± 14.8 years, including 9801 males (61.3%) and 6195 females (38.7%). More detail of baseline characteristics summarized in S1 Table as reported before. Further, baseline characteristics for the eligible and ineligible groups were shown in the Table 1. The proportion of individuals meeting eligible screening candidates was 35.87% (5737), with an average age of 58.75 ± 7.0 years. Among the eligible screening candidates, the proportion of males was higher than females (65.12% vs. 34.88%). Additional results are shown in Table 1.

**Table 1:** Baseline characteristics of the study subjects in eligible screening candidates and ineligible screening candidates.

	Eligible screening candidates (n = 5737)	Ineligible screening candidates (n = 10,259)	Statistics	*P* value
Sex				
Male, n%	3736 (65.12%)	6065 (59.12%)	*χ*^2^ = 55.8635	< 0.0001
Female, n%	2001 (34.88%)	4194 (40.88%)		
Age at inclusion (years)^a^	58.75 ± 7.00	45.60 ± 15.89	*t* = 59.56	< 0.0001
Age group				
< 30	NA	1131 (11.02%)	*χ*^2^ = 3128.4434	< 0.0001
35–	NA	1024 (9.98%)		
40–	NA	1209 (11.78%)		
45–	NA	2669 (26.02%)		
50–	1988 (34.65%)	2674 (26.06%)		
55–	1516 (26.42%)	28 (0.27%)		
60–	947 (16.51%)	20 (0.19%)		
65–	651 (11.35%)	20 (0.19%)		
70–	635 (11.07%)	18 (0.18%)		
75–	NA	654 (6.37%)		
≥ 80	NA	785 (7.65%)		
Smoking status				
Smoking	1838 (32.04%)	2911 (28.38%)	*χ*^2^ = 23.6447	< 0.0001
Non-smoking	3899 (67.96%)	7348 (71.62%)		
Smoking volume (pack year)^a^				
< 10	182 (9.9%)	725 (24.91%)	*χ*^2^ = 687.0132	< 0.0001
10–	393 (21.38%)	695 (23.87%)		
20–	333 (18.12%)	509 (17.49%)		
30–	373 (20.29%)	116 (3.98%)		
40–	174 (9.47%)	51 (1.75%)		
50–	27 (1.47%)	18 (0.62%)		
≥ 60	73 (3.97%)	28 (0.96%)		
Unknown	283 (15.4%)	769 (26.42%)		
Family history of lung cancer				
Yes	164 (2.86%)	237 (2.31%)	*χ*^2^ = 4.5287	0.0333
No	5573 (97.14%)	10,022 (97.69%)		
Chronic lung disease^b^				
Yes	1824 (68.21%)	4854 (52.69%)	*χ*^2^ = 442.2437	< 0.0001
No	3913 (31.79%)	5405 (47.31%)		

^a^Values are presented as mean ± SD (range). ^b^Chronic lung diseases include the chronic obstructive pulmonary disease, diffuse pulmonary fibrosis, history of pulmonary tuberculosis and other respiratory diseases.

### Lung cancer screening results

The total number of positive screen was 6779 as reported before^17^. A total of 180 patients (2.66%) had lung biopsy reports. Pathology results from lung biopsy revealed 154 cancers (true-positive) and 26 benign results (false-positive), the total surgical false-positive biopsy rate was 4.17%, and ineligible group (7.69%) exhibited a significantly higher surgical false positive rate than eligible group (2.47%), P < 0.05. The detection rate of positive screening, biopsy rate, the false-positive biopsy rate and the detection rate of lung cancer in eligible screening candidates were all higher than ineligible screening candidates (Table 2).

**Table 2:** Lung cancer screening results.

	All (n = 15,996)	Eligible screening candidates (n = 5737)	Ineligible screening candidates (n = 10,259)	Statistics	*P* value
Total positive screening	6779 (42.40%)	2774 (48.35%)	4005 (39.04%)	*χ*^2^ = 130.7103	< 0.0001
Biopsy rate					
Total	180/6779 (2.66%)	100/2774 (3.60%)	80/4005 (2.00%)	*χ*^2^ = 30.6850	< 0.0001
Surgical biopsy	120/6779 (1.77%)	81/2774 (2.92%)	39/4005 (0.97%)	*χ*^2^ = 28.6518	< 0.0001
Trans-thoracic needle biopsy	41/6779 (0.60%)	18/2774 (0.65%)	23/4005 (0.57%)		
Image guided bronchoscopy biopsy	19/6779 (0.28%)	1/2774 (0.04%)	18/4005 (0.45%)		
False-positive biopsy rate					
Total	26/180 (14.44%)	15/100 (15%)	11 (13.75%)	*χ*^2^ = 0.0562	0.8126
Surgical biopsy	5/120 (4.17%)	2/81 (2.47%)	3/39 (7.69%)*	*Fisher*	0.1398
Trans-thoracic needle biopsy	16/41 (39.02%)	5/18 (27.78%)	11/23 (47.83%)*		
Image guided bronchoscopy biopsy	5/19 (26.32%)	1/1 (100%)	4/18 (22.22%)*		
The cumulative detection rate of lung cancer	154/6779 (0.96%)	85/2774 (3.06%)	11/4005 (0.27%)	*χ*^2^ = 25.2581	< 0.0001

*The bonferrioni post-test was used for further pairwise comparison, *P* < 0.05.

### Gender, age and smoking status characteristics of lung cancer detected

**Figure 1:**
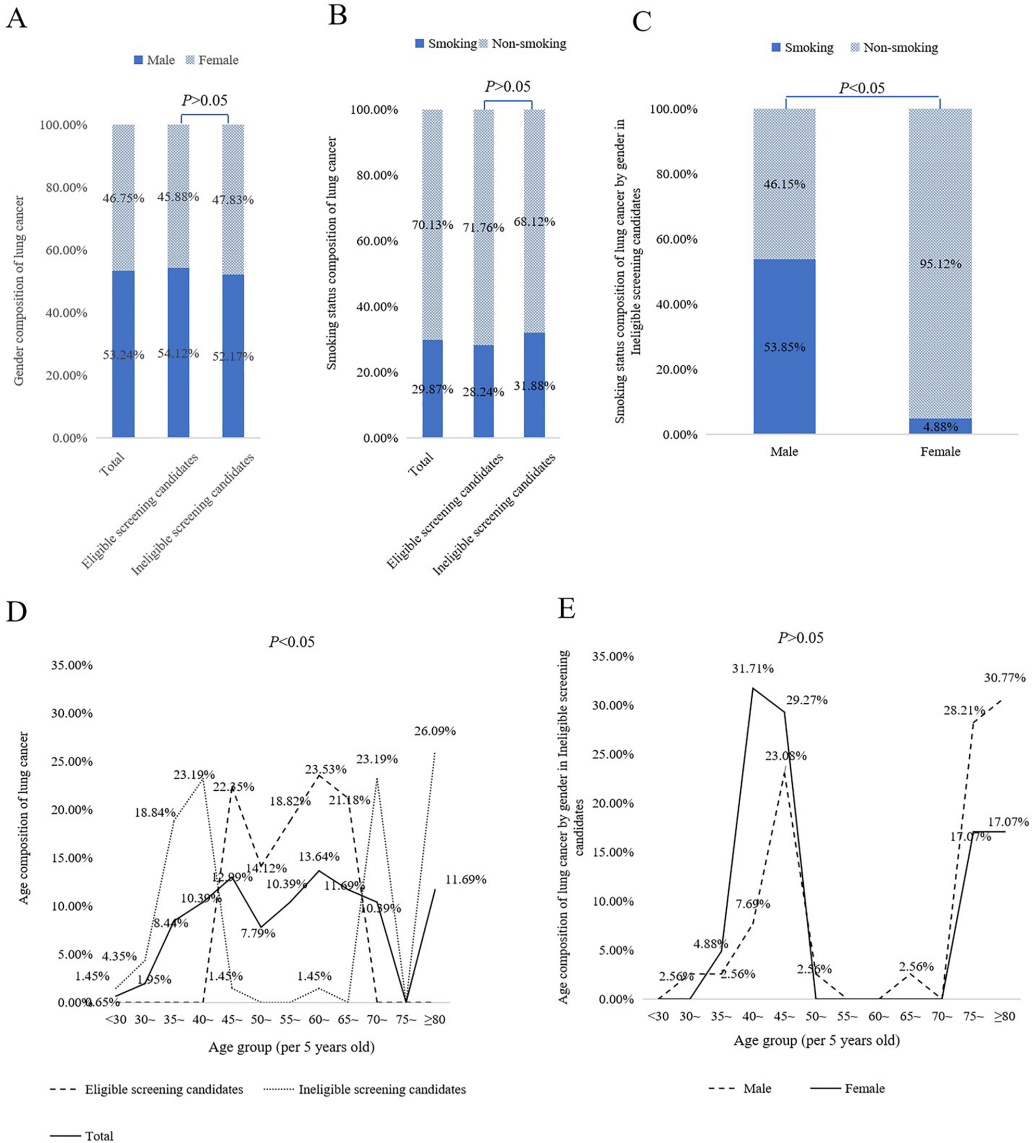
Gender, age and smoking status characteristics of lung cancer detected.

Among the 154 lung cancer patients, 82 (53.24%) were male and 72 (46.75%) were female. There was no statistical significant difference in gender between eligible and ineligible groups (Fig. 1A). The proportion of lung cancer cases in current smokers (29.87%) was lower than never smoked group (70.13%), and there was no statistically significant difference in smoking status between the two groups (Fig. 1B). Further, in ineligible group, the proportion of current smokers was higher among males compared to females (53.85% vs. 4.88%, P < 0.05) (Fig. 1C). The population was divided into 12 age groups with a 5-year span in age. In ineligible group, the age distribution of lung cancer cases showed two double peaks, 40–44 years old group (23.19%) and ≥ 80 years old group (26.09%) respectively (Fig. 1D). Further analysis by gender, the age distribution of lung cancer cases’ two double peaks was different in males and females (Males: 45–49 years old group (23.08%), ≥ 80 years old group (30.77%) vs. Females: 40–44 years old group (31.71%), ≥ 75 years old group (17.07%)) (Fig. 1E).

### The pathological classification and stage of lung cancer detected

**Figure 2:**
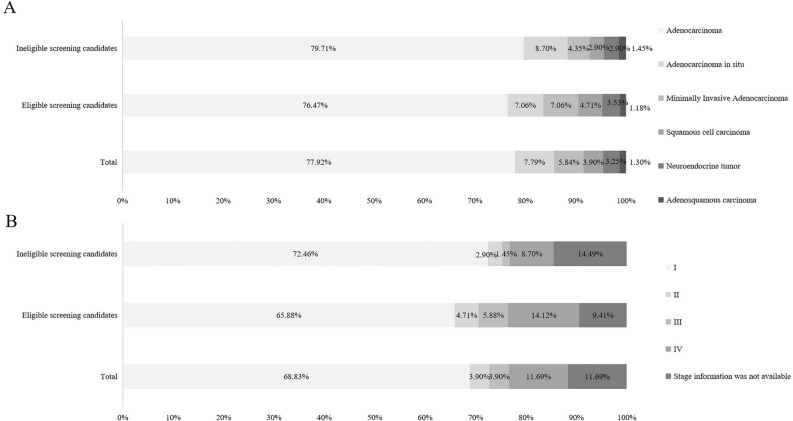
The pathological classification and stage of lung cancer.

Of the 154 lung cancer patients detected in this study, there were 120 cases of adenocarcinoma, 12 cases of adenocarcinoma in situ, 2 cases of adenosquamous carcinoma, 9 cases of minimally invasive adenocarcinoma, 5 cases of neuroendocrine tumor, and 6 cases of squamous carcinoma. Lung adenocarcinoma accounts for a high proportion of lung cancers both in ineligible group (79.71%) and eligible group (76.47%). More results were showed in Fig. 2A. Pathologic stages were stage I in 106 cases, stage II in 6 cases, stage III in 6 cases, stage IV in 18 cases, and 18 cases stage information were not available. Lung adenocarcinoma accounts for a high proportion of lung cancers both in ineligible group (79.71%) and eligible group (76.47%). More results were showed in Fig. 2B.

### Gender, and smoking status characteristics of pathological classification and stage of lung cancer in ineligible screening candidates

**Figure 3:**
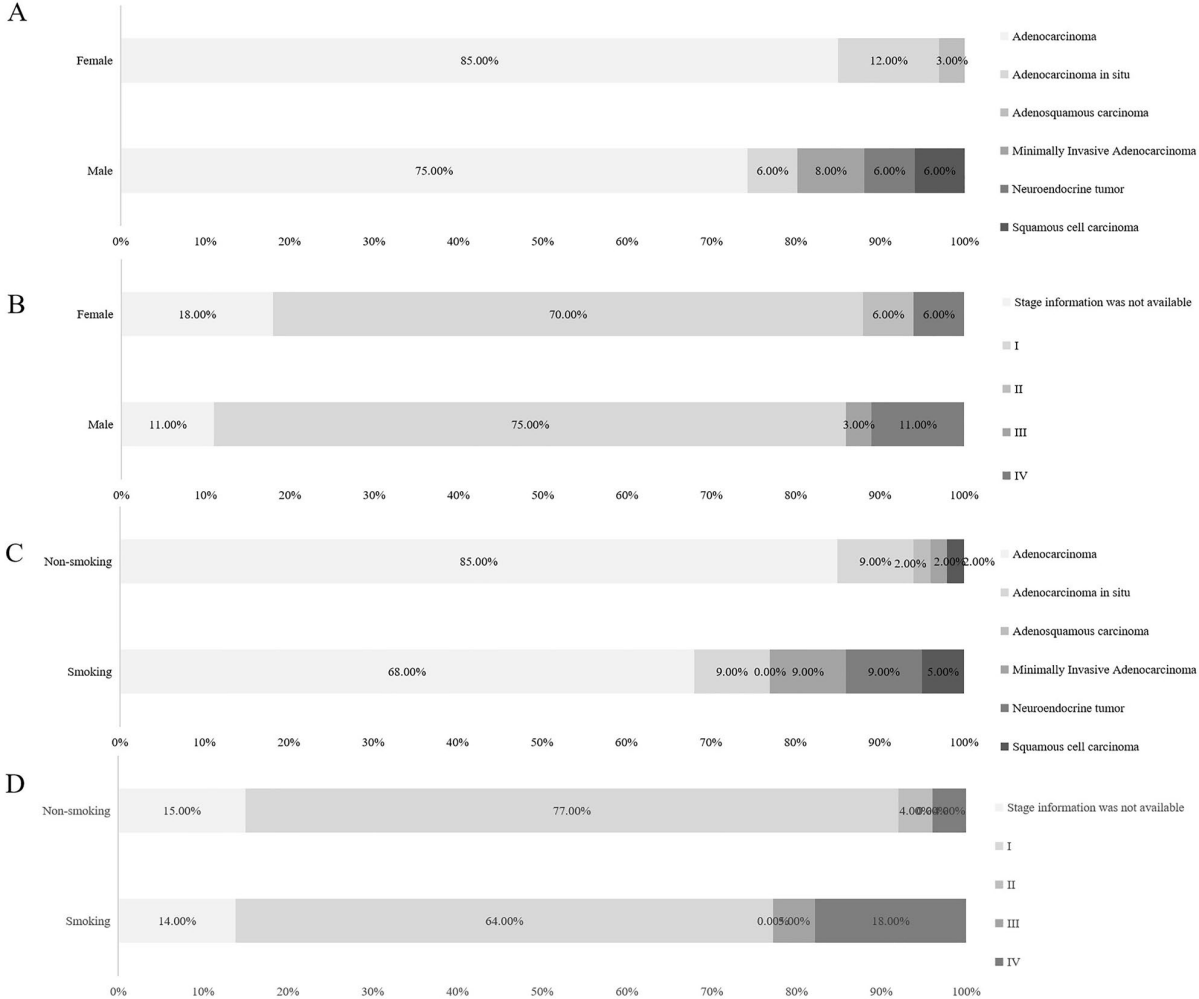
Gender, and smoking status characteristics of pathological classification and stage of lung cancer in ineligible screening candidates.

We further conducted a subgroup analysis of lung cancer case characteristics by gender and smoking status among ineligible group. 69 patients with lung cancer were identified in ineligible group, lung adenocarcinoma accounts for a high proportion of lung cancers both in male (75.00%) and female (85.00%). Squamous cell carcinoma was detected only in male (6.00%), but not in female. More results were showed in Fig. 3A. Whether male or female, pathological staging is dominated by stage I (Fig. 3B). Lung adenocarcinoma proportions are higher in non-smoking group (85.00%) than in smoking group (68.00%) (Fig. 3C). The pathological staging of lung cancer in smoking group and non-smoking group was mainly stage I, and the proportion of stage IV in male (18.00%) was higher than in female (2.00%). More results were showed in Fig. 3D.

## Discussion

In the present study, we investigated the LDCT screening results in ineligible candidates. The proportion of ineligible screening candidates was as high as 64.13%. This rate is similar to that reported by others^11,18^. A retrospective study of Asian lung cancer patients treated at New York reported that the percentage of Asian patients meeting the NLST criteria is similar to the United States population^19^. Although the overall rate of ineligibility for screening was similar to the rates reported previously, its internal composition was indeed quite different from previously reported. We found that the proportion of current smokers in males was almost ten times compared to females in ineligible screening candidates. At present, many studies have shown that we should pay enough attention to female and non-smokers^20^. Matthew Koshy analyzed differences in gender sensitivity of USPSTF screening guidelines that are based solely on age and smoking history, they found that the sensitivities for screening by the USPSTF 2013 were 46.7% for women and 64.6% for men and by the USPSTF 2021 were 56.8% and 71.8%, respectively^21^. The results of a large cross-sectional study showed that the odds of eligibility were lower for women compared with men (adjusted odds ratio, 0.88; 95% CI, 0.79–0.99) by the current revised USPSTF 2021^22^. Nearly two-thirds of lung cancer patients were non-smokers and an additional one-thirds were smokers, comprised mostly of female patients. The most important change in the newly revised LDCT screening eligibility guidelines in China was that lung cancer risk factors besides smoking were considered for the identification of high-risk population, mitigating the exclusion of female non-smokers from potential screening benefits^13,14^. A study about epidemiology of lung cancer and lung cancer screening programs in China and the United States revealed that the incidence of lung cancer in non-smokers that was significantly higher in China than in the United States, and was particularly notable in female^23^. Tony Kirby reported a young non-smoker diagnosed with lung cancer that who believed the system should be improved in many ways, from raising awareness in doctors and the public, removing stigma, and expanding screening for those who are excluded by the current screening guidelines^24^.

Our results are important for another reason. As the results shown in Table 2, the cumulative detection rate of lung cancer in ineligible screening candidates (0.27%) was lower than in eligible screening candidates (3.06%). It would appear that the yield of CT screening in ineligible subjects has a yield of diagnosis of lung cancer of less than 10% of that in eligible subjects. Clearly, such results may increase the challenges for recruiting female non-smokers into the screening process. According to current lung cancer screening guidelines, high-risk groups are defined according to factors such as age and smoking^4–7^. China’s lung cancer screening guidelines define high-risk groups in terms of passive smoking, smoking, family medical history, occupational exposure, history of chronic lung disease, and so on. However, it seems that factors other than age and smoking, there is lack of universal and unified other factors quality evaluation standards. The data in this study was derived from real-world data. China has not yet clarified the best age range for screening. Taking into account the cost and benefit of screening, the current recommended age range for screening is between 50 and 74 years old. With the promotion of lung cancer screening and the improvement of medical awareness, the onset of lung cancer has showed a younger trend^16^. However, there are significant differences in the study populations and the cost-effectiveness of supplemental screening for female non-smokers has yet to be evaluated. Prospective, multi-center or community-derived research support is still needed.

In practice, we also want to know whether there was a high false-positive rate for surgical pathology biopsies among ineligible candidates? It was surprising to find no differences in the false-positive biopsy rate between ineligible and eligible groups. Despite this, we were not yet confident in extrapolating this result. Of the 80 biopsy positive cases in ineligible group, there were 11 false negative diagnoses (5 surgical excision, 2 rans-thoracic needle biopsy and 4 image guided bronchoscopy). LDCT lung cancer screening has been shown to significantly reduce lung cancer mortality, but also more false-positive results, unnecessary testing and invasive procedures, overdiagnosis, etc.^25,26^. The proportion of invasive procedures following a false positive also increased significantly from 0.7 to 2.0% in the National Lung Screening Trial^27^. Our results found that the total population surgical false-positive biopsy rate was 4.17%, and the ineligible group (7.69%) was higher than the eligible group (2.47%). This would suggested that the surgical false-positive biopsy rate would likely be higher in a real-world setting, especially in ineligible candidates from health check-ups population. In 2019, West China Hospital of Sichuan University launched a full-process management project for pulmonary nodules/lung cancer patients, which could enables a standard follow-up screening procedure and treatment system^28^. All individuals aged 40 and older who participated in LDCT health check-ups in West China Hospital could received the green channel for diagnosis and treatment. This will provide an opportunity to gain more evidence about the cost-effectiveness of LDCT screening in ineligible candidates, female non-smokers in particular. Note that besides relying on current treatment guidelines, there are also other methods for selecting high-risk candidates. Pan-Canadian Early Detection of Lung Cancer (PanCan) Study selected participants for lung cancer screening by risk modeling^29^. A risk-based predictive model based on family history of lung cancer and female gender may improve lung cancer screening efficiency, according to a Taiwanese Lung Cancer Screening Project study^30^. Lung cancer screening using computed tomography can reduce mortality, but it is also critical to optimize the balance of benefits and risks by increasingly selecting high-risk groups. At present, more and more studies have confirmed that screening high-risk groups of lung cancer based on individualized risk is a very effective way to participate in LDCT screening, even better than guideline standards^31^.

The present study has some additional limitations. Firstly, there were insufficient data available regarding incidence of mortality and long-term lung cancer morbidity during the study periods included in this analysis. Secondly, the prevalence of smoking in the health check-ups female population presented in our database was low (4.88%), further research incorporating more female smokers is needed to serve as a control group. Also, whether our findings apply to more ethnically diverse populations awaits further study.

## Conclusion

The proportion of ineligible screening candidates and the surgical false-positive biopsy rate in ineligible candidates were both high in health check-ups population.

## Methods

### Study design and participants

**Figure 4:**
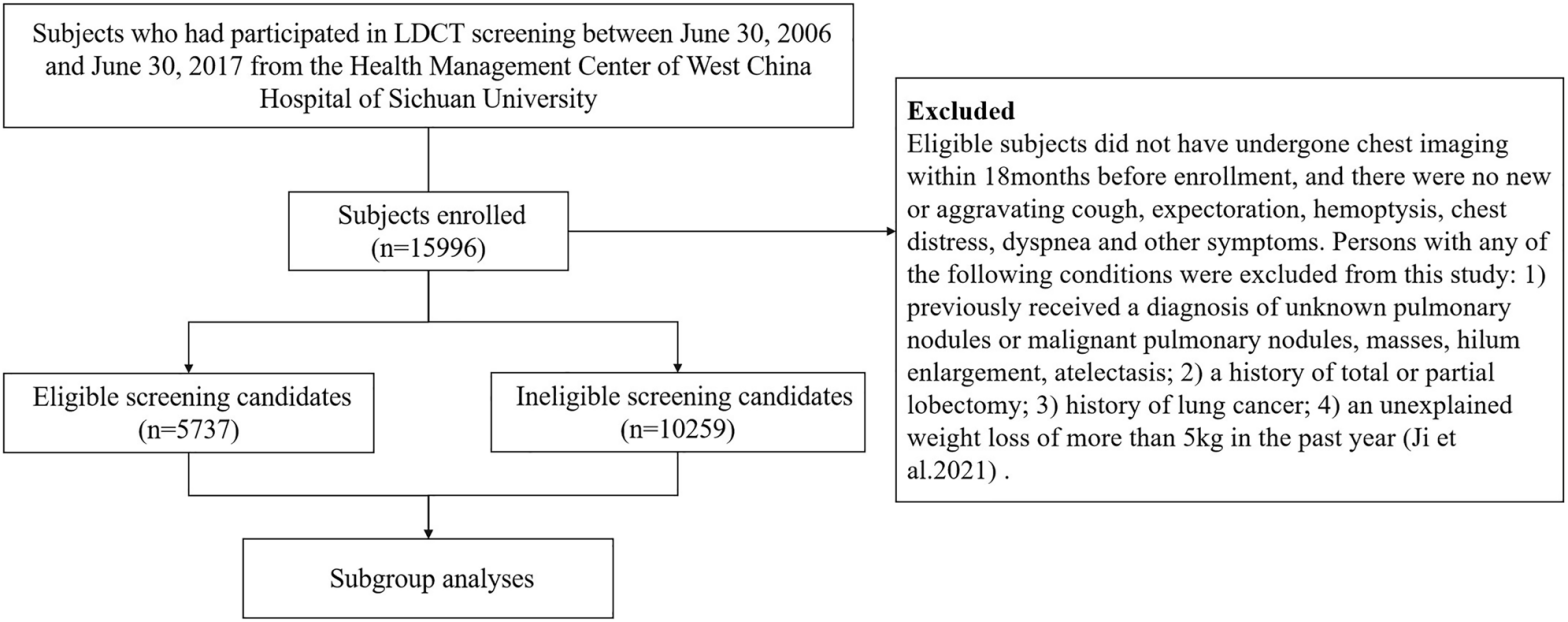
Study flow diagram.

The study was a retrospective analysis, we have used data from a real-world study of low-dose computed tomography screening among people who took yearly health checkup. More details about the study design, entry criteria, screening protocol, and medical-record abstraction of this study have been previously published^17^. Baseline characteristics were showed in S1 Table. According to the eligible criteria, a total of 15,996 subjects were eventually included (Fig. 4).

### Positive screening criteria

The criteria for a positive screen was according to the China National Lung Cancer Screening Guideline with Low-dose Computed Tomography (2018 version)^13^, a positive screen was defined as any of the following: (1) baseline screening: any of the following: (a) with a diameter of ≥ 5 mm pure or part-solid nodule; (b) any nonsolid nodules ≥ 8 mm; (c) Suspicious bronchial lesion or (and) tracheal lesion; (d) single or multiple nodules or lung cancer masses diagnosed as lung cancer by LDCT. (2) annual screening: any of the following: (a) discovery of new noncalcific nodules or airway lesions; (b) it is found that the original nodule is enlarged or the solid component is increased.

### Ethical consideration

Before collecting data, this research had been approved by the Biomedical Ethics Review Committee of West China Hospital of Sichuan University (2015(202), 2019(195)). Informed consent for the study was obtained from each study participant and/or their legal guardian(s) before data collection and documented in a prepared format. Moreover, all methods in the present study were performed in accordance with the declarations of Helsinki and the relevant guidelines/regulations.

### Follow-up time and vital status

The follow-up was a part of the routine procedure as recommended by the treating physician. We have a working mechanism for early warning of major positive diseases and a green channel (Specialized outpatient clinic for lung cancer, convenient for follow-up and standardized diagnosis and treatment). This study were followed-up from January 1, 2007 to June 1, 2022. The shortest follow-up time was 3.5 years, and the longest was 15.5 years. As previously reported^17^, we have trained professionals to follow-up. A special follow-up team is responsible for ascertaining probable vital status and determining whether the cause of death was lung cancer. We also evaluated harms’ outcomes, specifically false-positive biopsy. Lung cancer pathological typing was according to the International Association for the Study of Lung Cancer (IASLC) Eighth Edition of the TNM Classification for Lung Cancer^32^.

### Grouping criteria

We used China National Lung Cancer Screening Guideline with Low-dose Computed Tomography (2018 version) as ineligible candidates grouping basis^13^. Eligible screening candidates: (a) aged 50–74 years, (b) who have at least a 20 pack-year smoking history, (c) and who currently smoke or have quit within the past 5 years. Ineligible screening candidates: the above conditions were not met.

### Statistical analysis

Statistical analyses were performed using SPSS software (SPSS statistics 21.0; SPSS Inc.). The comparison of the continuous variable was analyzed using a two-sided *t*-test and the categorical variables was analyzed using a Chi-square testing or Fisher exact test. The Bonferroni post-test was used for further pairwise comparison. Considering the comparisons of age trends, linear trend Chi-square test had been applied. The test level α = 0.05 was considered statistically significant.

### Supplementary Information

Supplementary Table S1.

## Data Availability

Data are not publicly available but may be accessed upon reasonable request from the corresponding author (huairongtang1963@163.com).
