# Supplementary material for: LDCT screening results among eligible and ineligible screening candidates in preventive health check-ups population: a real world study in West China
Source: Sci Rep. 2024 Feb 28;14:4848. doi: 10.1038/s41598-024-55475-x (PMC10902338; doi:10.1038/s41598-024-55475-x)
Supplement: Supplementary file 1 — Supplementary Table S1. [file 41598_2024_55475_MOESM1_ESM.docx]

Supplementary Material

# Supplementary Tables

Supplementary Table1 Baseline characteristics

| Parameter | Male, n (%) | Female, n (%) | Total, n (%) |
| --- | --- | --- | --- |
| Total | 9801 (61.3) | 6195 (38.7) | 15,996 (100.0) |
| Age at inclusion (years) ^a^ | 51.26 ± 14.76 | 48.80 ± 14.76 | 50.31 ± 14.81 |
| < 30 | 545 (5.56) | 586 (9.5) | 1131 (7.0) |
| 30~ | 1361 (13.9) | 872 (14.1) | 2233 (14.0) |
| 40~ | 3119 (31.8) | 2224 (35.9) | 5343 (33.4) |
| 50~ | 2363 (24.1) | 1196 (19.3) | 3559 (22.2) |
| 60~ | 1033 (10.5) | 605 (9.8) | 1638 (10.2) |
| 70~ | 832 (8.5) | 475 (7.7) | 1307 (8.2) |
| ≥80 | 548 (5.6) | 237 (3.8) | 785 (5.0) |
| Smoking status |  |  |  |
| smoking | 4697 (47.9) | 52 (0.8) | 4749 (29.7) |
| non-smoking | 5104 (52.1) | 6143 (99.2) | 11,247 (70.3) |
| Smoking volume (pack year) ^a^ | 19.24 ± 14.84 | 13.95 ± 25.04 | 19.20 ± 14.93 |
| 10 | 894 (19.0) | 13 (25.0) | 907 (19.1) |
| 10~ | 1080 (23.0) | 8 (15.4) | 1088 (23.0) |
| 20~ | 841 (17.9) | 1 (1.9) | 842 (17.7) |
| 30~ | 488 (10.4) | 1 (1.9) | 489 (10.3) |
| 40~ | 225 (4.8) | 0 (0) | 225 (4.7) |
| 50~ | 45 (1.0) | 0 (0) | 45 (0.9) |
| ≥60 | 100 (2.1) | 1 (1.9) | 101 (2.1) |
| Unknown | 1024 (21.8) | 28 (53.8) | 1052 (22.2) |
| Family history of lung cancer |  |  |  |
| Yes | 187 (1.91) | 214 (3.45) | 401 (2.5) |
| No | 9614(98.09) | 5981 (96.55) | 15,595 (97.5) |
| Chronic lung disease ^b^ |  |  |  |
| Yes | 5868 (59.9) | 3450 (55.7) | 9318 (58.3) |
| No | 3933 (40.1) | 2745 (44.3) | 6678 (41.7) |

a Values are presented as mean ± SD (range). b Chronic lung diseases include the chronic obstructive pulmonary disease, diffuse pulmonary fibrosis, history of pulmonary tuberculosis and other respiratory diseases.
